# Psychometric properties of the schizotypal personality questionnaire-brief revised (SPQ-BR) in a German-speaking sample

**DOI:** 10.1038/s41598-026-38037-1

**Published:** 2026-02-18

**Authors:** Vida Gajic, Marie Fitzner, Theresa Schulze, Paul Klusmann, Lyn Giemsa, Phillip Grant, Salome Becker, Inge Maria Hahne, Marco Zierhut, Stephan Ripke, Alice Braun, Julia Kraft

**Affiliations:** 1 Department of Psychiatry and Psychotherapy, Charité-Universitätsmedizin Berlin, Berlin, Germany; 2 German Center for Mental Health (DZPG), Partner Site Berlin/Potsdam, Berlin, Germany; 3 Stanley Center for Psychiatric Research, Broad Institute of MIT and Harvard, Cambridge, MA USA; 4 Berlin Institute of Health at Charité-Universitätsmedizin Berlin, BIH Biomedical Innovation Academy, BIH Charité Clinician Scientist Program, Berlin, Germany; 5 Division of Psychopathology and Psychosis Research, Psychology School, Faculty of Health and Social Sciences, Fresenius University of Applied Sciences, Frankfurt am Main, Germany

**Keywords:** Diseases, Health care, Medical research, Neuroscience, Psychology, Psychology

## Abstract

Schizotypy refers to personality traits linked to an increased risk for schizophrenia-spectrum disorders (SSD). The Schizotypal Personality Questionnaire-Brief Revised (SPQ-BR) is a widely used 32-item self-report measure, but its psychometric properties in German-speaking populations are unexplored. This study comprised 738 individuals, including 33 with SSD, and 148 with other mental disorders. Participants (mean age: 38 years, 77% female) completed an online survey that included the German version of the SPQ-BR (SPQ-BR-G) and additional measures to assess convergent and discriminant validity. Reliability, latent factor structure, and validity were assessed. The SPQ-BR-G demonstrated excellent internal consistency (α = 0.91), good convergent validity (r = 0.52–0.58), and discriminant validity (r < 0.50) for the cognitive-perceptual and disorganized factors. Confirmatory factor analysis supported three- and four-factor solutions, while a single-factor model demonstrated poor fit. Subjective well-being was negatively associated with the SPQ-BR-G, after adjusting for sex, age, and education. Compared to the survey sample, SSD patients scored significantly higher on all factor levels and most items (p < 0.05), with 35% ranking in the top SPQ-BR-G decile. In SSD patients, moderate to high correlations were observed between the negative (r = 0.86, p < 0.001) and positive (r = 0.53, p < 0.05) dimensions of the Positive and Negative Syndrome Scale and the SPQ-BR-G. The SPQ-BR-G shows robust psychometric properties, supporting its use in schizotypy research. Its validation enhances cross-cultural comparisons and may aid in early risk and biological risk factor identification for SSD.

**Supplementary Information:**

The online version contains supplementary material available at 10.1038/s41598-026-38037-1.

## Introduction

Schizotypy encompasses a cluster of personality traits associated with psychosis and schizophrenia-spectrum disorders (SSD). It spans a continuum from subtle, subclinical traits in the general population to more pronounced characteristics in individuals with SSD. Schizotypy is multidimensional, including positive, negative, and disorganized facets that correspond with related symptom dimensions in SSD^1–4^.

Schizotypal (Personality) Disorder (SPD) is considered one of the various clinical manifestations of schizotypy, characterized by persistent symptoms, significant distress^5,6^, and considerable functional impairment, although typically less disabling than schizophrenia^7^. The conceptualizations of Schizotypal (Personality) Disorder between the DSM-V^8^ and the ICD-10^9,10^ differ slightly. The DSM describes only a stable personality disorder, while the ICD allows for a shorter symptom manifestation of at least 2 years possibly accompanied by transient (pseudo-)psychotic experiences. These ICD-defined features overlap with those observed in individuals at ultra-high risk for psychosis. Identifying individuals at risk for such disorders is a key priority in mental health research. Over the past 40 years, various screening tools have been developed to assess schizotypy, often leveraging instruments designed for screening for Schizotypal (Personality) Disorder, so-called schizotypal traits^11^. Measures primarily include self-report scales, alongside interviews assessing specific symptoms or the broader range of schizotypal traits. Prominent scales for measuring schizotypy include the Oxford-Liverpool Inventory of Feelings and Experiences (O-LIFE), the Wisconsin Schizotypy Scale (WSS)^11–13^, and the Multidimensional Schizotypy Scale (MSS)^14^, while schizotypal traits are commonly assessed using the Schizotypal Personality Questionnaire (SPQ)^15^. The SPQ is often also used as a proxy for the measurement of schizotypy, even though the concepts are not completely interchangeable^11^.

The SPQ, initially developed by Raine^15^ to screen for Schizotypal (Personality) Disorder, has also become a popular tool for capturing schizotypal traits within the general population, measuring both normal and abnormal degrees of schizotypy^16^. This self-report questionnaire consists of 74 forced-choice items across nine scales derived from the DSM-III-R criteria for SPD^17^: Ideas of Reference, Social Anxiety, Magical Thinking, Unusual Perceptual Experiences, Paranoid Ideation, Lack of Close Friends, Constricted Affect, Odd Behavior, and Odd Speech^15,18^. Cohen et al. introduced an updated version of the SPQ, called the Schizotypal Personality Questionnaire-Brief Revised (SPQ-BR)^18^. This version reduces the questionnaire to 32 items, rated on a 5-point Likert scale, providing enhanced response options and greater sensitivity. The SPQ-BR has been validated in English, Spanish, and Hungarian populations^18–20^.

Although the SPQ and its brief version, SPQ-B, have been utilized in German-speaking populations^19,21,22^, psychometric properties of the SPQ-BR have yet to be assessed. Here, we present a comprehensive validation study that includes assessments of reliability, scale structure, and validity of the SPQ-BR in a German population sample and a clinical sample of individuals with SSD.

## Results

### Description of the case and survey sample

A total of 986 individuals initially started the online survey. Of these, 268 were excluded due to incomplete SPQ-BR data. Among the remaining participants, 11 were excluded for providing zero-variance responses, meaning they showed equal ratings across all SPQ-BR items. Additionally, two individuals were excluded due to zero-variance responses in other questionnaires, and irrelevant answers in open-text response sections in combination with implausible demographic data. The final dataset consisted of 705 participants who completed the SPQ-BR, with 656 (93%) of the participants completing the entire survey (see Supplementary Fig. 1). Participants had a mean age of 38 (SD = 12), with the majority (77%) identifying as female.

Detailed demographic characteristics, including education, employment status, family status, and housing status, for the survey sample and the case sample, are presented in Table 1. The “case sample” included 33 participants, of whom 21 were diagnosed with schizophrenia and 12 diagnosed with schizoaffective disorder.

**Table 1:** Sociodemographic characteristics of the survey and case sample.

Characteristic	Survey sample, N = 705^a^	Case sample, N = 33^a^
Age^a^	38 (12)	39 (13)
Gender^b^		
Male	164 (23%)	19 (59%)
Female	539 (77%)	12 (38%)
Native language (German)^b^	627 (89%)	27 (82%)
Education (ISCED—97)^b^		
Lower secondary	6 (0.9%)	5 (16%)
Upper secondary	211 (30%)	14 (44%)
Post-secondary non-tertiary	145 (21%)	4 (13%)
First stage tertiary	290 (41%)	8 (25%)
Second stage tertiary education	53 (7.5%)	0 (0%)
Employment^b^		
Employed, full-time	438 (62%)	3 (9.7%)
Employed, part-time	187 (27%)	4 (13%)
In training/studying	106 (15%)	7 (23%)
Other	74 (11%)	22 (67%)
Relationship status^b^		
In a relationship/married	458 (66%)	12 (40%)
Single/divorced/widowed	240 (34%)	18 (60%)
Housing^b^		
Living alone	185 (26%)	15 (50%)
Living with someone	517 (73%)	15 (50%)
Family history of mental illness (positive)^b^	201 (29%)	–
Self-reported history of mental illness (positive)^b^	148 (21%)	–
Current residence^b^		
Berlin	593 (84%)	–
Other	112 (16%)	–

^a^Mean (*SD*); ^b^*n* (%).

### Sex differences

No significant differences between male and female participants were observed for the SPQ-BR total score or the three superordinate factors (p > 0.05, Supplementary Table 2). At the subordinate factor level, small but significant differences were observed for Eccentric Behavior (p < 0.001) and Magical Thinking (p < 0.001), with men scoring higher on Eccentric Behavior (M_male_ = 4.69, SD = 3.54; M_female_ = 3.67, SD = 3.53) and women higher on Magical Thinking (M_male_ = 2.50, SD = 3.51; M_female_ = 3.43, SD = 3.82).”

### Reliability

The SPQ-BR demonstrated excellent internal consistency for the full scale, with a Cronbach’s alpha of 0.91 and McDonald’s omega of 0.93. The alpha value was 0.85-0.86 for all three superordinate factors, and omega values ranged from 0.89 (for *Cognitive-Perceptual* and *Interpersonal*) to 0.92 (for *Disorganized*). Reliability estimates for the seven individual scales are detailed in Table 2, with alpha values ranging from 0.75 (*Unusual Perception*) to 0.88 (*Odd Speech and Social Anxiety*) and omega values ranging from 0.79 (*Unusual Perception*) to 0.90 (*Magical Thinking* and *Odd Speech*).

**Table 2:** Reliability estimates and Spearman correlations between SPQ-BR scales (N = 705).

Scale	Reliability		SPQ-BR correlations					
	α	ω	IR/S	CF/CA	EB	SA	MT	OS
SPQ-BR total	0.91	0.93						
Superordinate factors								
Cognitive-perceptual	0.85	0.89						
Interpersonal	0.85	0.89						
Disorganized	0.86	0.92						
Subordinate factors								
Ideas of reference/suspiciousness (IR/S)	0.79	0.84						
No close friends/constricted affect (CF/CA)	0.77	0.81	0.43***					
Eccentric behavior (EB)	0.86	0.88	0.42***	0.45***				
Social anxiety (SA)	0.88	0.89	0.42***	0.52***	0.46***			
Magical thinking (MT)	0.86	0.90	0.33***	0.12**	0.20***	0.16***		
Odd speech (OS)	0.88	0.90	0.41***	0.22***	0.41***	0.29***	0.24***	
Unusual perception (US)	0.75	0.79	0.42***	0.29***	0.40***	0.32***	0.42***	0.39***

*p < 0.05, **p < 0.01, ***p < 0.001.

α = Cronbach’s alpha; ω = McDonald’s omega.

### Scale structure

Next, we conducted a confirmatory factor analysis (CFA) examining one-factor, three-factor, and four-factor solutions of the SPQ-BR. Both the three-factor and four-factor models demonstrated satisfactory fit (CFI = 0.99, TLI = 0.98, RFI = 0.98, RMSEA = 0.06, SRMR = 0.06). The chi-square difference test comparing the four-factor model (χ^2^ = 1471.06, df = 453) to the three-factor model (χ^2^ = 1472.11, df = 454) showed χ^2^(1) = 03, p = 0.58, indicating no significant difference between the two models. In contrast, the one-factor model displayed a notably poorer fit [CFI = 0.84, TLI = 0.83, RFI = 0.82, RMSEA = 0.19, SRMR = 0.15, χ^2^ = 11,864.38 (df = 464)] than the four- and three-factor models. Both models fit the data significantly better than the one-factor model, indicated by the chi-square difference tests (one-factor vs. three-factor: χ^2^(10) = 1998.1, p < 0.001; one-factor vs. four-factor: χ^2^(11) = 1998.4, p < 0.001). The goodness-of-fit indices of all examined models are summarized in Table 3.

**Table 3:** Goodness-of-fit indices for three CFA models (N = 705).

Model	χ^2^	df	CFI	TLI	RMSEA	SRMR	RFI
1-Factor model	11,864.38	464.00	0.84	0.83	0.19	0.15	0.82
3-Factor model	1472.11	454.00	0.99	0.98	0.06	0.06	0.98
4-Factor model	1471.06	453.00	0.99	0.98	0.06	0.06	0.98

χ^2^, Chi-square; df, degrees of freedom; CFI, comparative fit index; TLI, Tucker–Lewis index; RMSEA, root mean square error of approximation; SRMR, standardized root mean square residual; RFI, relative fit index.

**Fig. 1:**
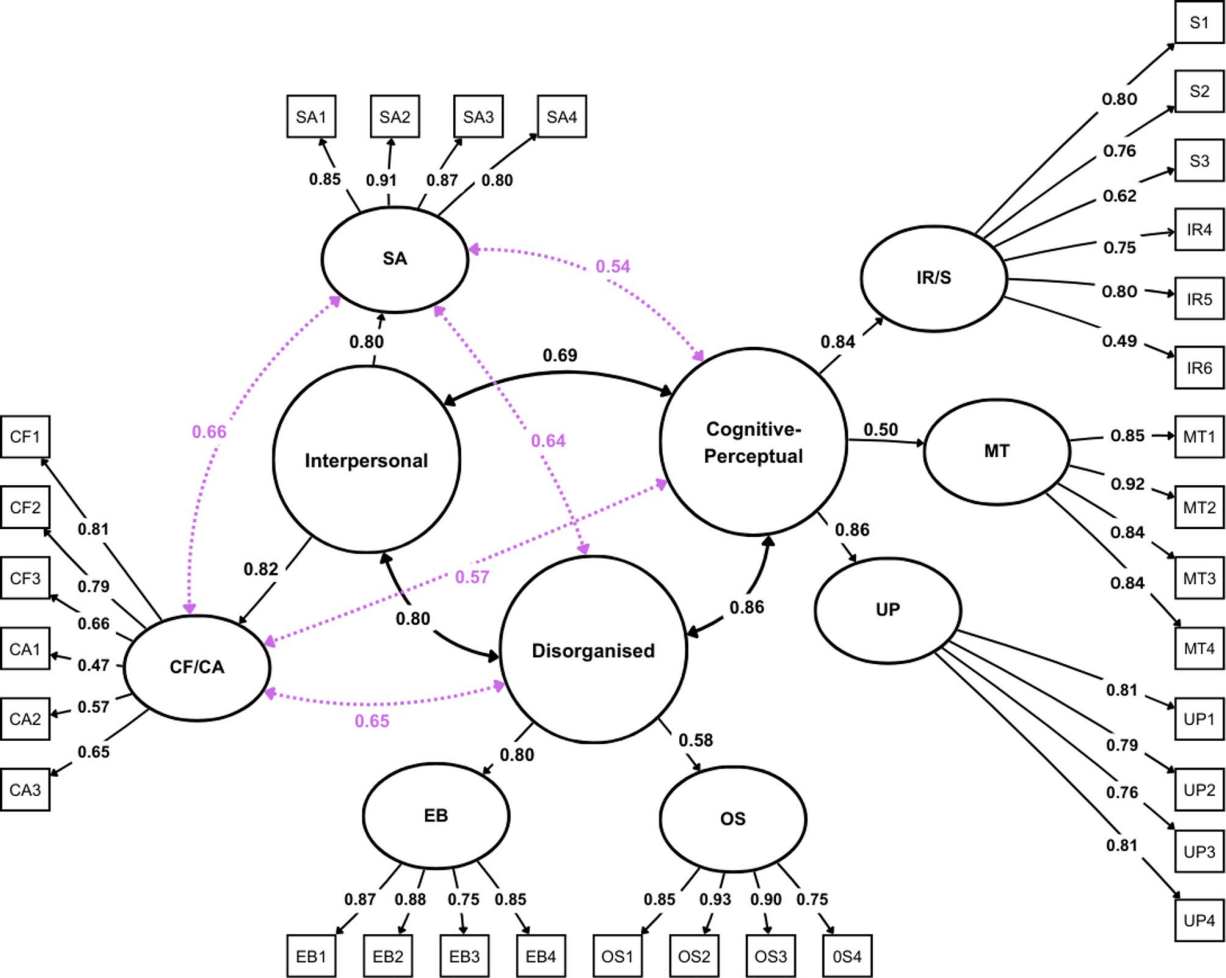
The three-factor superordinate structure of the revised SPQ-BR (N = 705). Numbers at bidirectional paths indicate covariance between latent factors, while unidirectional paths show standardized factor loadings. For comparison, the four-factor superordinate structure is depicted with blue dashed lines for visual distinction. All factor loadings remain identical across both models. IRS, Ideas of reference/suspiciousness; MT, Magical thinking; UP, Unusual perception; CF, No close friends; CA, Constricted affect; SA, Social anxiety; EB, Eccentric behaviour; OS, Odd speech.

A path diagram including item factor loadings and factor covariances of the three- and four-factor models is shown in Fig. 1. Factor loadings for all items across the three-factor and four-factor models were above 0.4, suggesting adequate item contributions to their underlying latent factors, with loadings ranging from 0.47 to 0.93. All scales correlated significantly with their respective superordinate factors, with factor loadings ranging from r = 0.5 (*Magical Thinking*) to r = 0.86 (*Unusual Perception*).

### Validity

#### Correlation with schizotypy, psychotic symptoms, and other constructs

Convergent and discriminant validity of the SPQ-BR were assessed through correlations with related and unrelated constructs, as summarized in Table 4. All SPQ-BR superordinate factors demonstrated statistically significant correlations with relevant scales of the Oxford-Liverpool Inventory of Feelings and Experiences Short (sO-LIFE) and SCL-90-R (p < 0.001). For instance, the *Cognitive-Perceptual* factor strongly correlated with the sO-LIFE *Unusual Experiences* scale (r = 0.58, p < 0.001) and the Symptom Checklist-90-Revised (SCL-90-R) *Paranoid Ideation* scale (r = 0.55, p < 0.001). When the SCL-90-R items are used to compose scores for Schizophrenia Nuclear Symptoms (SNS) and Schizotypal Signs (STS), we observe high correlations with STS (r = 0.55, p < 0.001) and moderate correlations with SNS (r = 0.34, p < 0.001). Similarly, the *Disorganized* SPQ-BR factor showed strong correlations with the sO-LIFE *Cognitive Disorganization* scale (r = 0.52, p < 0.001). A moderate correlation was observed between the SPQ-BR *Interpersonal* factor and sO-LIFE *Introvertive Anhedonia* (r = 0.48, p < 0.001) and *Cognitive Disorganization* (r = 0.48, p < 0.001) scales.

**Table 4:** Spearman correlations of SPQ-BR superordinate factors with the sO-LIFE, SCL-90-R, NEO-FFI-30, and PHQ-4 scales (N = 656).

	Cognitive-perceptual	Interpersonal	Disorganized
sO-LIFE			
Unusual experience	0.58***	0.23***	0.41***
Introvertive anhedonia	0.19***	0.48***	0.31***
Cognitive disorganization	0.40***	0.48***	0.52***
Impulsive nonconformity	0.29***	0.15***	0.33***
SCL-90-R			
Paranoid ideation	0.55***	0.44***	0.45***
Psychoticism	0.45***	0.49***	0.46***
Schizophrenia nuclear symptoms	0.34***	0.24***	0.25***
Schizotypal signs	0.55***	0.52***	0.50***
NEO-FFI-30			
Neuroticism	0.44***	0.55***	0.46***
Extraversion	− 0.05	− 0.47***	− 0.06
Openness to experience	0.07	− 0.02	0.16***
Agreeableness	− 0.33***	− 0.39***	− 0.40***
Conscientiousness	− 0.15***	− 0.19***	− 0.28***
PHQ-4			
Anxiety	0.35***	0.40***	0.35***
Depression	0.32***	0.43***	0.35***

*p < 0.05, **p < 0.01,***p < 0.001.

sO-LIFE, Oxford-Liverpool Inventory of Feelings and Experiences; SCL-90-R, Symptom Checklist 90 revised; NEO-FFI-30, NEO Five-Factor Inventory; PHQ-4, Patient Health Questionnaire 4.

Among the NEO Five-Factor Inventory-30 (NEO-FFI-30) traits, *Neuroticism* displayed moderate to high correlations with all SPQ-BR superordinate factors (r = 0.44–0.55), particularly with the *Interpersonal* factor (r = 0.55, p < 0.001). *Extraversion* showed significant negative correlations with the *Interpersonal* factor (r =  − 0.47, p < 0.001). *Conscientiousness* and *Agreeableness* exhibited significant negative correlations with all three SPQ-BR superordinate factors (r = − 0.40 to − 0.15), suggesting that higher schizotypal traits are associated with lower *Conscientiousness* and *Agreeableness*.

Correlations with the Patient Health Questionnaire-4 (PHQ-4) anxiety and depression subscales were moderate (r = 0.32–0.43, p < 0.001), indicating a relation between schizotypal traits and general mental distress.

Notably, the SPQ-BR *Social Anxiety* (SA) scale showed a stronger correlation with *Neuroticism* (r = 0.54, p < 0.001) than the *No Close Friends/Constricted Affect* (CF/CA) scale (r = 0.42, p < 0.001). Similarly, the SA scale correlated more strongly with PHQ-4 Anxiety (r = 0.42, p < 0.001) than CF/CA (r = 0.29, p < 0.001). A detailed comparison of correlations with SPQ-BR scales is provided in Supplementary Table 3.

#### Association with well-being

Association between SPQ-BR scores and subjective well-being as measured by the PWI-A were examined in regression models. Sum scores of the full SPQ-BR scale showed significant negative associations with the PWI-A composite score (β = − 0.43, 95% CI [− 0.49, − 0.36], p < 0.001) as well as all three superordinate factors: *Cognitive-Perceptual* (β = − 0.61, 95% CI [− 0.75, − 0.47]), *Interpersonal* (β = − 1.07, [− 1.22, − 0.92]), and *Disorganized* (β = − 0.91, 95% CI [− 1.11, − 0.72]), all p < 0.001.

Each of the seven PWI-A domains also exhibited significant negative associations with the SPQ-BR (see Supplementary Table 4), indicating that higher schizotypal traits, as measured by the SPQ-BR, are consistently linked to lower life satisfaction across various domains, including health, social participation, and personal relationships.

#### Family history of psychosis and other mental disorders

We further examined differences in SPQ-BR total, superordinate, and scale scores of individuals with and without a family history of psychosis or other mental disorders. As shown in Supplementary Table 5, there were no significant differences in SPQ-BR total scores between those with a positive family history of psychosis (M = 35.94, SD = 20.19) and those without (M = 35.61, SD = 18.04; p > 0.9). Similarly, no significant differences were observed across superordinate factors or scales (p > 0.5).

Likewise, no significant differences were found in SPQ-BR total scores, superordinate factors, or scales (see Supplementary Table 5) between individuals with a negative versus positive family history of other mental disorders (p > 0.2).

#### History of mental illness and SSD

We also examined differences in SPQ-BR total and scale scores between individuals with no history of mental illness, those with SSD, and those with a history of other mental disorders (see Supplementary Table 6).

**Fig. 2:**
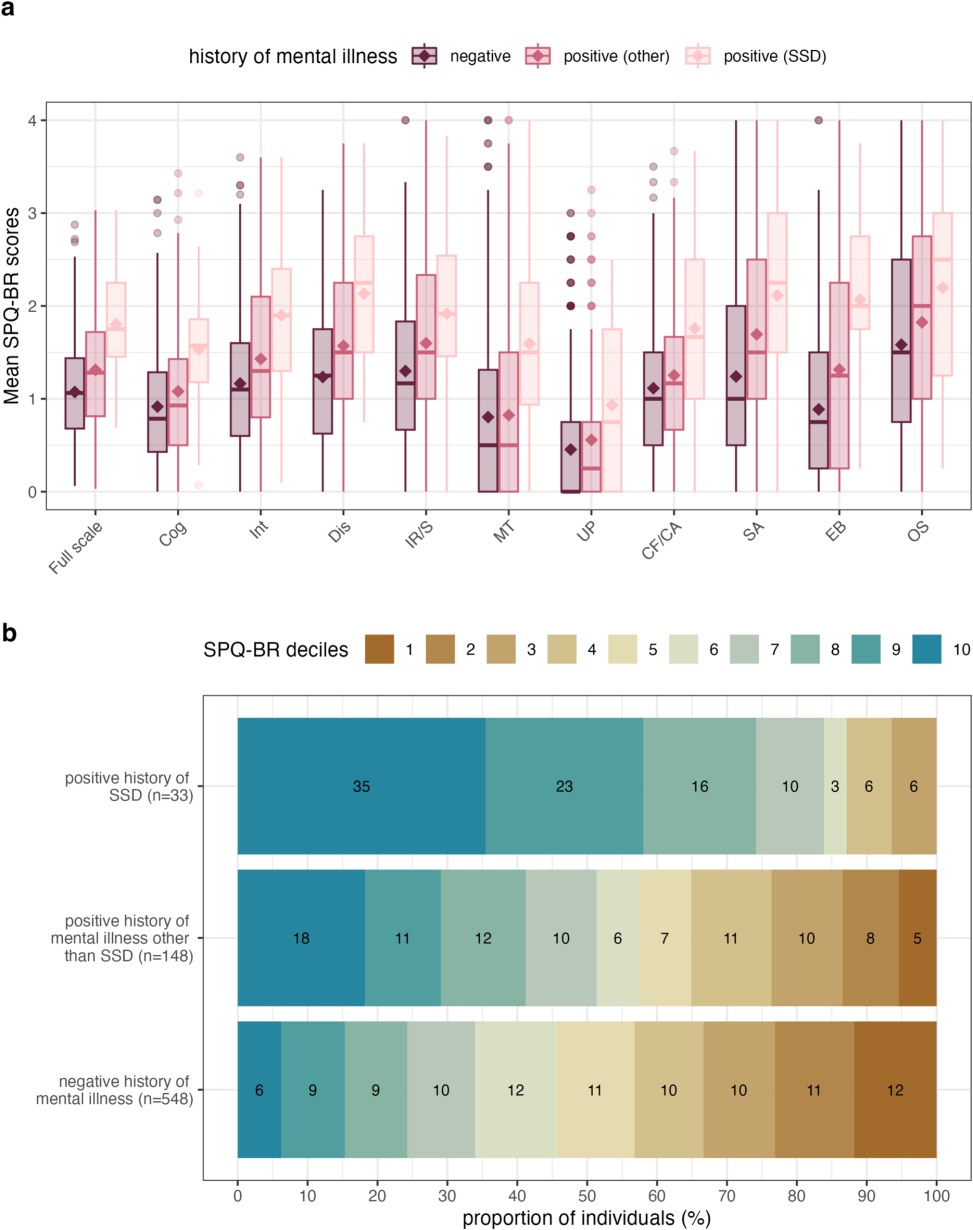
Differences in SPQ-BR scores between individuals with a negative history of psychiatric illness (n = 548), a positive history of schizophrenia/schizoaffective disorder (n = 33) and other psychiatric disorders (n = 148). (**a**) Boxplots of mean SPQ-BR scores. Diamonds represent mean values for each group. Cog, Cognitive-Perceptual; Int, Interpersonal; Dis, Disorganized; IR/S, Ideas of reference/suspiciousness; MT, Magical thinking; UP, Unusual perception; CF, No close friends; CA, Constricted affect; SA, Social anxiety; EB, Eccentric behaviour; OS, Odd speech. (**b**) Barplot illustrating the proportion of individuals (%) in each SPQ-BR decile (full scale) per group.

The distributions of SPQ-BR scores for all three groups are visualized in Fig. 2a. Individuals with SSD showed the highest SPQ-BR total scores (M = 58, SD = 20), followed by individuals with other mental disorders (M = 42, SD = 21), and individuals without any history of mental illness (M = 34, SD = 17). Differences in SPQ-BR total scores were significantly higher in the case sample than in the survey sample, which includes individuals with and without other mental disorders (p < 0.001). In the survey sample, total SPQ-BR scores for participants without any history of mental illness were significantly lower than those affected by a mental disorder other than SSD (p < 0.001).

A decile analysis, visualized in Fig. 2b, highlighted that 35% of individuals with SSD (n = 11) were in the top SPQ-BR decile compared to only 9% of the overall survey sample and 6% of individuals without a history of mental illness. In the survey sample, 18% of the individuals affected by another mental disorder scored in the top SPQ-BR decile (see Fig. 2b). The SPQ-BR score ranges per decile are reported in Supplementary Table 7.

Despite significant differences in SPQ-BR total scores, superordinate factors and scales, as well as some items, showed minimal differences between the SSD and survey sample, including the items S2, CA1, OS1, and UP4 (see Supplementary Table 6 for detailed comparisons).

#### Exploring relationships between SPQ-BR and PANSS in cases with SSD

Finally, we explored relationships between SPQ-BR superordinate and Positive and Negative Syndrome Scale (PANSS) factor scores in the case sample using pairwise Spearman correlations. The highest correlation between the SPQ-BR *Interpersonal* factor was observed with the PANSS negative factor (r = 0.86, p < 0.001). The SPQ-BR *Cognitive-Perceptual* factor was positively correlated with both the PANSS positive (r = 0.53, p < 0.05) and PANSS depressive (r = 0.53, p < 0.05) factors. The SPQ-BR *Disorganized* factor was highly correlated with PANSS negative (r = 0.70, p < 0.01) and PANSS hostility (r = 0.54, p < 0.05) factors but not the cognitive-disorganized PANSS factor (r = 0.09, p > 0.05; see Supplementary Table 8).

## Discussion

This study examined the psychometric properties of the SPQ-BR, a measure of schizotypal traits, within a German-speaking population. The results show good reliability, comparable to the English and the Spanish version^19,23^. CFA results supported both three- and four-factor models, with minor variations, mirroring findings of the original study by Cohen^18^. These findings suggest that the SPQ-BR-G captures the multidimensionality of schizotypal traits, supporting previous research^24,25^.

While our results support convergent validity between the positive (*Unusual Experience*) and disorganized (*Cognitive Disorganization*) scales of the sO-LIFE and the *Cognitive-Perceptual* and *Disorganized* SPQ-BR dimensions, we only found a moderate correlation (r < 0.5) between the negative sO-LIFE scale *Introvertive Anhedonia* and the *Interpersonal* factor of the SPQ-BR. This is in line with previous findings that of the three factors, *Interpersonal* shares the least variance with “true” (negative) schizotypy; instead tapping more into *Neuroticism*^3,11,26^. This is not unexpected, however, as the SPQ was not intended to capture “true” schizotypy, but rather screen for potential SPD risk; thus, it is based on a slightly different theoretical framework^4^.

Differences in these scales may reflect varying conceptualizations of “negative symptoms” (of SPD) vs “negative schizotypy”. The sO-LIFE assesses a broader range, sharing variance with Introversion, while the SPQ taps primarily into social anhedonia and adds a slight aspect of perceived distress. Also, while social anxiety is reflected in both measures to some extent, it is an explicit scale of the SPQ (loading onto *Interpersonal*), while in the sO-LIFE, it is primarily a function of the suboptimal discriminatory power of some of the items from the *Cognitive Disorganisation* scale^3^. Past investigations reported similar relationships between these factors, with correlations between sO-LIFE *Introvertive Anhedonia* and SPQ-BR *Interpersonal* ranging between 0.4 and 0.67^19,27^. Interestingly, the SPQ-BR *Interpersonal* factor also showed a high correlation with the negative symptom factor of the PANSS, which is in agreement with previously observed correlations between the original PANSS negative scale and SPQ *Interpersonal* factor^28^.

In addition to positive dimensions measured by other schizotypy instruments, our findings also indicate convergent validity of the SPQ-BR’s *Cognitive-Perceptual* factor and state-like psychotic measures, particularly with the subscales *Paranoid Ideation* and *Schizotypal Signs* of the SCL-90-R. This is in line with previous findings showing significant correlations between SPQ and SCL-90-R subscales and indicates the SPQ-BR’s ability to assess stable schizotypal traits as well as more recent symptomatology.

As expected, SPQ-BR factors showed significant correlations with the *Neuroticism* scale due to the slight overlap of the traits that these scales measure^23,29^. For instance, the SPQ-BR assesses difficulties in interpersonal functioning, whilst aspects of neuroticism, such as hostility and impulsiveness, also contribute to interpersonal troubles^30^. The negative correlations found between the *Interpersonal* factor, comprising social anxiety, and *Extraversion*, generally associated with outgoingness^29^, aligns with earlier findings of the German SPQ^22^.

In line with previous research, our findings corroborate an inverse relationship between schizotypy and subjective well-being^18,31^. Other lines of research suggest the existence of a subgroup (“happy schizotypes”), referring to individuals with high levels of positive schizotypy and simultaneously low levels of negative and disorganized schizotypy, who experience higher creativity^32^, greater well-being^33^, and good mental health^34,35^. Such findings could be a result of individual differences in the samples, the measurement tools used, or the multifaceted nature of schizotypy itself^36^ or simply the finding that such individuals appear to be extremely rare^37^.

Cross-cultural differences in schizotypal traits are increasingly being studied, demonstrating that cultural variations can impact the expression and perception of schizotypy^19,38–40^. The present study, conducted in a German-speaking population, adds a new cultural perspective to this body of research. For example, in comparison to Spanish and U.S.-American populations^19^, schizotypy expression in our data more closely resembled that of the Spanish-speaking sample. This further highlights the importance of interpretative caution when comparing SPQ-BR scores across cultural settings and the necessity of future research examining cultural differences as well as the potential influences of language on item difficulty^41^.

Past research has shown higher scores in individuals with a positive compared to a negative family history of schizophrenia on the SPQ-BR’s *Cognitive-Perceptual* factor and its scales, *Magical Thinking* and *Ideas of Reference/Suspiciousness*^42^. Contrary to this, we do not observe more frequent “positive” schizotypal traits in individuals with a positive family history of psychosis or other psychiatric disorders. Considering that only six participants reported a family history of schizophrenia, we used family history of a broader psychosis phenotype. Therefore, a direct comparison with these findings is likely hampered by the inconsistent definitions. In line with previous SPQ findings^28,43^, we observed consistently higher schizotypal traits in individuals with SSD compared to unaffected individuals, supporting the SPQ-BR-G’s group validity.

Despite the adequate psychometric properties of the SPQ-BR-G, there are some limitations to our study that need to be considered. Firstly, the exploratory correlation analysis between the SPQ-BR and the PANSS factor scores in the SSD group may not be representative due to the small sample size. Investigating how schizotypal traits align with schizophrenia symptoms is crucial for improving screening tools. Addressing this would require longitudinal study designs to determine the predictive value of SPQ-BR ratings over time. As our study did not include clinically diagnosed SPD cases, further validation, e.g., using structured clinical interviews, may be needed to establish criterion validity. Secondly, we mainly relied on existing literature to assess the internal scale structure. Although three- and four-factor models seem plausible in light of our data, alternative models could offer an equally good or better fit. More recent research found that SPQ-BR items are best represented by a theoretical structure of nine lower-order factors, with bifactor models also showing adequate goodness-of-fit indices^19^. Thirdly, as our survey was mainly distributed in Berlin, this sample may not fully reflect the diversity of the broader German-speaking population.

In conclusion, our study provides robust evidence for the validity and reliability of the SPQ-BR in German-speaking populations. Establishing instruments that are valid and reliable in a range of populations may facilitate future research that increases our understanding of schizotypal traits, its relation to mental health, and biological underpinnings of symptoms across the psychosis spectrum.

## Methods

### Participants

Our study included two distinct samples: (1) a non-clinical group that participated in an online survey (“survey sample”), (2) a clinical sample consisting of patients with a lifetime diagnosis of schizophrenia or schizoaffective disorder according to ICD-10 criteria (“case sample” with SSD) was recruited through the “Berlin Research Initiative for Diagnostics, Genetics, and Environmental Factors in Schizophrenia” (BRIDGE-S)^44^. Proceedings of the BRIDGE-S study are described elsewhere in detail^44^.

The survey sample was drawn from the general population and primarily recruited online, with invitations distributed via university email lists, intranet postings, and social media channels. Participants were included if they were at least 18 years old and possessed sufficient German language skills to complete the online survey. To enhance response rates, a 15€ local supermarket voucher was raffled among a random selection of 25 participants.

### Data collection and instruments

#### The questionnaire catalogue

The survey presented to the non-clinical participants comprised seven questionnaires beginning with demographic items, including age, gender, education level, and questions about the participants’ and their families’ mental health histories. Subsequently, participants completed a series of standardized self-report measures, including the SPQ-BR, the sO-LIFE, the NEO-FFI-30, the SCL-90-R subscales *Psychoticism* and *Paranoid Ideation*, the PHQ-4, and the Personal Wellbeing Index for adults (PWI-A) to capture schizotypy and schizotypal traits, quality of life, as well as other personality traits and mental health symptoms. At the end of the survey, participants answered questions from the Alcohol, Smoking and Substance Involvement Screening Test (ASSIST). The online survey was implemented in a Research Electronic Data Capture platform (REDCap)^45^ hosted by the Charité.

#### The Schizotypal Personality Questionnaire Brief Revised (SPQ-BR)

The SPQ-BR is a 32-item scale developed by Cohen et al.^18^ as an enhanced version of the original Schizotypal Personality Questionnaire (SPQ)^15^. The SPQ-BR uses a five-point Likert scale ranging from *“strongly disagree”* ("trifft gar nicht zu") to *“strongly agree”* ("trifft vollkommen zu"). The higher-order factor structure consists of three dimensions: *Cognitive-Perceptual* (14 items), *Interpersonal* (10 items), and *Disorganized* (8 items), that tap into the positive, negative, and disorganized facets of schizotypy to some extent^3,11^. The German version, based on Klein et al.’s^22^ translation, was refined for this study with minor updates to grammar and spelling. Additionally, the wording of item six was modernized to align with contemporary German language usage. The German SPQ-BR questionnaire version is included in the Supplementary Table 1.

#### Oxford-Liverpool Inventory of Feelings and Experiences Short (sO-LIFE)

The sO-LIFE is a tool designed for the measurement of schizotypy in the general population via four scales: *Unusual Experiences* (positive schizotypy), *Cognitive Disorganization* (disorganized schizotypy), *Introvertive Anhedonia* (negative schizotypy), and *Impulsive Nonconformity* (a fourth scale included to assess a broader spectrum of traits in line with the notion of unitary psychosis)^46^. The short version contains 43 dichotomous (*yes/no*) items, including several reverse-coded items on the *Introvertive Anhedonia* and *Impulsive Nonconformity* factors, and shows good reliability (α = 0.68–0.88) across both English and German samples^41,46^.

#### Symptom Checklist-90-Revised (SCL-90-R)

The SCL-90-R assesses a variety of mental distress symptoms experienced within the past seven days^47^. For this study, two subscales of the SCL-90-R were selected: *Paranoid Ideation* (6 items), assessing hostility, suspiciousness, and paranoid thoughts^48^, and *Psychoticism* (10 items)*,* measuring symptoms from mild estrangement to severe psychotic experiences^49^. Both subscales are rated on a 5-point Likert scale ranging from “*not at all*” to *“extremely*”.

Furthermore, we computed sum scores for the *Schizotypal Signs* (STS, 8 items) and *Schizophrenia Nuclear Symptoms* (SNS, 4 items) dimensions. STS captures distress related to interpersonal difficulties, paranoid ideation, and suspiciousness, while SNS assesses distress caused by symptoms such as thought-broadcasting and thought-intrusions, delusions of control, and auditory hallucinations^50^. These dimensions were derived from factor analyses^51^ and are based on selected items from the *Paranoid Ideation* and *Psychoticism* subscales of the SCL-90-R.

#### NEO Five-Factor Inventory-30 (NEO-FFI-30)

The NEO-FFI-30 captures individual differences across five key personality dimensions: *Neuroticism*, *Extraversion*, *Openness*, *Agreeableness*, and *Conscientiousness*. It is based on the Five-Factor Model, a theoretical model developed by Costa and McCrae in 1989^52^. The short version consists of 30 items rated on a 5-point Likert scale, ranging from *“strongly disagree”* to *“strongly agree”*. The inventory is commonly used in personality research, is time-efficient, and shows sufficient reliability (α = 0.61—0.85). It also demonstrated factor-, convergent- (r = 0.40—0.56) and discriminant validity (r = (-0.21)—(-0.56)) in a German sample^53^.

#### Patient Health Questionnaire for Depression and Anxiety (PHQ-4)

The PHQ-4 is an ultra-short screening tool for depression and anxiety, comprising two items each for depression (PHQ-2) and anxiety (GAD-2)^54^. It uses a 4-point Likert scale, ranging from *“not at all”* to *“almost every day”*, and demonstrates acceptable internal consistency (α = 0.75—0.82) and discriminant validity (r = (-0.28)—(-0.49)) in a German-speaking sample^55^.

#### Personal Wellbeing Index—Adult (PWI-A)

The PWI-A measures subjective well-being through eight questions covering life satisfaction and specific domains such as standard of living, health, achievements in life, relationships, safety, community-connectedness, and future security. Each domain is represented by one item, scored on an 11-point scale (ranging from 0 to 10)^56^. It has good psychometric properties, including convergent validity (r = 0.78) and internal consistency (α = 0.80—0.90). The instrument has been translated into various languages, including German^57^.

### Statistical Analysis

#### Reliability

To assess the reliability of the SPQ-BR, we calculated Cronbach’s alpha and McDonald’s omega for the entire scale, its three superordinate factors, and seven subordinate factors, using the *psych package* (Version 2.4.6)^58^ in R (version 4.3.0.). Acceptable reliability was defined as alpha values between 0.70 and 0.95^59^ and McDonald’s omega values of 0.7 and above^60^. The test–retest reliability was explored in a small, independent sample and is reported in the Supplementary Material.

#### Scale Structure

Previous literature suggests a multi-dimensional factor structure of the SPQ-BR^18,42^. A CFA was conducted in the survey sample to test three models: a one-factor model, a three-factor model (*Cognitive-Perceptual*, *Interpersonal*, and *Disorganized* superordinate factors), and a four-factor model (that splits the Interpersonal superordinate factor into *No Close Friends and Constricted Affect* and *Social Anxiety*). In both the three- and the four-factor models, the *Cognitive-Perceptual* factor comprises the scales *Ideas of Reference/ Suspiciousness, Magical Thinking,* and *Unusual Perception*, while the *Disorganized* factor includes *Eccentric Behavior* and *Odd Speech*. The *Interpersonal* factor in the three-factor model combines *No Close Friends/Constricted Affect* and *Social Anxiety*, whereas the four-factor model treats them separately^18^. The three- and four-factor model has previously been reported by Cohen et al.^18^. The WLSMV estimator was used due to the ordinal scale of the questionnaire items. Model fit was assessed using Comparative Fit Index (CFI ≥ 0.95), Tucker-Lewis Index (TLI ≥ 0.95), Root Mean Square Error of Approximation (RMSEA ≤ 0.06), Standardized Root Mean Square Residual (SRMR ≤ 0.08), and Relative Fit Index (RFI > 0.90)^61^. Satorra-Bentler scaled chi-square difference tests were conducted for model comparisons with a significance threshold set at α = 0.05. Results were visualized using path diagrams generated with the Software Ωnyx (Build number: 1.0–1026). Latent variables were scaled by fixing their variances to 1 to ensure identifiability. For all subsequent correlation and association analyses, standard item-based sum scores were computed according to the predefined superordinate and subordinate SPQ-BR factor structure.

#### Validity

#### Correlation with schizotypy, psychotic symptoms, and other constructs

Convergent and discriminant validity of the SPQ-BR were evaluated using Spearman correlations with overlapping measures (sO-LIFE, SCL-90-R *Psychoticism* and *Paranoid Ideation*) and distinct symptoms (PHQ-4), as well as personality traits (NEO-FFI-30). We used a threshold of r > 0.50 as evidence for convergent validity. The sub- and superordinate factors of the SPQ-BR were tested against the sO-LIFE, SCL-90-R, PHQ-4, and NEO-FFI-30 scales to evaluate convergence with specific dimensions.

#### Association with well-being

Associations between SPQ-BR sum scores of superordinate and subordinate factors with subjective well-being domains (PWI-A) were assessed using linear regression models adjusted for sex, age, and educational attainment. Effect sizes (beta), including 95% confidence intervals, and two-sided p-values are reported.

#### History of mental illness and family history

Group validity was assessed through comparisons of SPQ-BR scores at item, superordinate, subordinate, and scale level between a) individuals with vs. without self-reported mental disorders (within the survey sample), b) individuals with vs. without SSD (survey vs. case sample).

We further compared SPQ-BR scores of individuals with respect to a) a positive vs. negative family history of psychosis and b) a positive vs. negative family history of mental disorders other than schizophrenia or schizoaffective disorder. A family history of psychosis was defined as having a first- or second-degree relative diagnosed with schizophrenia, schizoaffective disorder, bipolar disorder, or other psychotic disorders.

Mean and standard deviation of SPQ-BR scores are reported for each group. Group comparisons were conducted at the item, subordinate, superordinate, and scale levels using t-tests and Wilcoxon rank-sum tests, as appropriate.

#### Exploring relationships between SPQ-BR and PANSS in cases with SSD

Lastly, we examined the relationship between SPQ-BR and the PANSS^62^, a broadly used, rating-based instrument measuring schizophrenia symptoms across three original domains: positive symptoms, negative symptoms, and general psychopathology. To examine the association between schizotypal traits and symptom severity in cases with SSD, we conducted pairwise Spearman correlations between SPQ-BR superordinate and PANSS positive, negative, cognitive-disorganized, depressive, and hostility factors^63^.

#### Missing data

The approach to handling missing data in this study was designed to maximize sample size and statistical power while minimizing the risk of bias. For descriptive analysis, reliability assessments, and factor analysis, all participants who completed the demographic section and the SPQ-BR questionnaire were included. Correlation and association analyses were conducted with participants who completed the entire survey. Furthermore, certain items regarding personal and family history were optional. Therefore, the N corresponds to the number of participants who provided responses to these items.

## Supplementary Information

Supplementary Information.

## Data Availability

The datasets generated and analyzed during the current study are available from the corresponding author upon reasonable request. Access to the data may be subject to a data transfer agreement and applicable ethical or institutional restrictions.
