# Supplementary material for: Psychometric properties of the schizotypal personality questionnaire-brief revised (SPQ-BR) in a German-speaking sample
Source: Sci Rep. 2026 Feb 18;16:7176. doi: 10.1038/s41598-026-38037-1 (PMC12920991; doi:10.1038/s41598-026-38037-1)
Supplement: Supplementary file 1 — Supplementary Information. [file 41598_2026_38037_MOESM1_ESM.docx]

# Table of Contents

**Supplementary Methods.**

**Supplementary Results.**

**Supplementary Discussion.**

**Supplementary Table 1**. German SPQ-BR questionnaire

**Supplementary Figure 1**. Survey sample workflow and exclusion criteria

**Supplementary Figure 2.** Differences in SPQ-BR scores across time

**Supplementary Table 2.** Differences in SPQ-BR factor, subscale and item scores between male and female participants (N=703)

**Supplementary Table 3**. Spearman correlations of SPQ-BR subordinate factors with the O-LIFE, SCL-90-R, NEO-FFI-30 and PHQ-4 scales (N=656)

**Supplementary Table 4**. Association between SPQ-BR and personal wellbeing (PWI-A) (N=656)

**Supplementary Table 5**. Differences in SPQ-BR factor, subscale and item scores between individual with/without a family history of psychosis and mental disorders

**Supplementary Table 6**. Differences in SPQ-BR factor, subscale and item scores between individuals with/without schizophrenia or schizoaffective disorder and other self-reported mental disorders

**Supplementary Table 7**. SPQ-BR total sum score ranges per decile in the survey sample (N=736)

**Supplementary Table 8.** Pairwise Spearman correlations between PANSS factor scores (Lim et al.) and SPQ-BR superordinate factors (N=33)

**Supplementary Table 9.** Sociodemographic characteristics of the independent, longitudinal sample (n=22)

**Supplementary Table 10**. Differences in SPQ-BR scores across time

**Supplementary References.**

**Supplementary Methods**

***Exploratory test-retest reliability and temporal stability of SPQ-BR ratings***

Test-retest reliability was explored using an independent sample of individuals without SSD recruited through the BRIDGE-S study[^1^](https://paperpile.com/c/2iIoZC/iB9s). As part of internal study quality control procedures, a random subset of participants were recruited twice into the study at random time points. Of these, 22 individuals completed the SPQ-BR twice. The participants were recruited via newsletters, public and university advertisements and the general population. Detailed study proceedings are described elsewhere. Intraclass correlation coefficients (ICCs) were calculated for the full scale to evaluate the consistency of scores across repeated assessments. We used ICC(3,k), a two-way mixed-effects model that measures consistency across multiple ratings[^2^](https://paperpile.com/c/2iIoZC/gFog). The ICC was calculated using the *psych* R package. Partial Pearson’s correlations were additionally computed to control for variability in the time intervals between ratings, using the *ppcor* package in R. To explore temporal patterns, SPQ-BR scores at T1 and T2 were summarized using mean and SD, range, and the average change in SPQ-BR sum score difference (ΔSPQ-BR: T2-T1) for the entire sample, as well as for participants with ≤ 1 year versus >1 year between assessments. Additional analyses were conducted to explore the relationship between ΔSPQ-BR and the time interval in days (Pearson’s correlation), and the interaction between Δtime and T1 SPQ-BR sum score in a linear regression model predicting T2 SPQ-BR sum scores.

**Supplementary Results**

***Exploratory test-retest reliability and temporal stability of SPQ-BR ratings***

Test-retest reliability for the SPQ-BR total score was evaluated in 22 individuals who completed the questionnaire twice. Sociodemographic characteristics are shown in Supplementary Table 9. The time interval between test administrations varied considerably, with a mean of 405 ± 443 days (range: 10 to 1624 days). Pearson’s correlation was r = 0.71 (95%CI [0.42, 0.87], p < 0.001), and the intraclass correlation coefficient (ICC3,k) was 0.83 (95%CI [0.60, 0.93], p < 0.0001), indicating good test-retest reliability. A partial Pearson’s correlation analysis accounting for different time intervals between ratings showed a high correlation between ratings (r_par_ = 0.77, p< 0.0001).

Next we assessed the temporal stability of SPQ-BR sum scores across time. Mean SPQ-BR score decreased by -5.46 points from T1 to T2 (see Supplementary Table 10), although the decline is larger in individuals where T1 and T2 are more than 1 year apart (mean decrease: -9.40) compared to those where both ratings occurred within a year (mean decrease: -2.17). Thus, we found a significant negative correlation between ΔSPQ-BR sum scores and days between ratings (r = -0.46, 95%CI [-0.74, -0.05], p < 0.05; Supplementary Figure 2). In a linear regression model, T1 SPQ-BR sum scores significantly predicted T2 SPQ-BR sum scores (β = 0.86, p < 0.001), but no significant interaction between T1 SPQ-BR sum score and Δ days interaction was observed (p = 0.36).

**Supplementary Discussion**

***Exploratory test-retest reliability and temporal stability of SPQ-BR ratings***

Although good values for test-retest reliability were observed, the sample size and variability in time differences between measurements for each pair limit the interpretability of these findings. Consequently, these results should be regarded as preliminary, and future studies are required to provide robust estimates of test-retest reliability using a systematic assessment at fixed time points. A modest decline in SPQ-BR scores over time was observed, particularly for participants with longer time intervals between assessments, which is in agreement with prior studies that indicated a decline in SPQ means over a period 2-4 year[^3,4^](https://paperpile.com/c/2iIoZC/zDe9+vZ1i). Nevertheless, future studies with larger samples are crucial for characterizing long-term trajectories of schizotypal traits as measured by the SPQ-BR and potential factors associated with temporal changes.

**Supplementary Table 1**. German SPQ-BR questionnaire

| English SPQ-BR items (Cohen et al., 2010) | *Im folgenden Abschnitt finden Sie eine Reihe von Feststellungen oder Fragen. Bitte lesen Sie sich jede Aussage durch und kreuzen Sie an, wie stark diese Ihre Persönlichkeit widerspiegelt.* | | | | | |
| --- | --- | --- | --- | --- | --- | --- |
|  | **SPQ-BR** Adapted from the German SPQ (Klein et al., 1997) | Trifft  gar nicht zu | Trifft eher nicht zu | Weder noch | Trifft eher zu | Trifft vollkommen zu |
| **Do you sometimes feel that people are talking about you? (IR4)** | 1. Haben Sie manchmal das Gefühl, dass andere über Sie reden? | 0 | 1 | 2 | 3 | 4 |
| **Do you sometimes feel that other people are watching you? (IR5)** | 2. Haben Sie manchmal das Gefühl, dass andere Menschen Sie beobachten? | 0 | 1 | 2 | 3 | 4 |
| **When shopping do you get the feeling that other people are**  **taking notice of you? (IR6)** | 3. Haben Sie während des Einkaufens das Gefühl, dass andere Menschen Notiz von Ihnen nehmen? | 0 | 1 | 2 | 3 | 4 |
| **I often feel that others have it in for me. (S1)** | 4. Ich habe oft das Gefühl, dass andere es auf mich abgesehen haben. | 0 | 1 | 2 | 3 | 4 |
| **Do you sometimes get concerned that friends or co-workers are**  **not really loyal or trustworthy? (S2)** | 5. Machen Sie sich manchmal Sorgen darüber, ob Freunde oder Kollegen wirklich redlich und vertrauenswürdig sind? | 0 | 1 | 2 | 3 | 4 |
| **Do you often have to keep an eye out to stop people from**  **taking advantage of you? (S3)** | 6. Müssen Sie oft darauf achtgeben, dass andere Sie nicht ausnutzen? | 0 | 1 | 2 | 3 | 4 |
| **Do you feel that you cannot get “close” to people. (CF1)** | 7. Haben Sie das Gefühl, dass sie mit anderen Menschen nicht „warm“ werden? | 0 | 1 | 2 | 3 | 4 |
| **I find it hard to be emotionally close to other people. (CF2)** | 8. Ich finde es schwierig, einen engen emotionalen Kontakt zu anderen Menschen zu haben. | 0 | 1 | 2 | 3 | 4 |
| **Do you feel that there is no one you are really close to outside of**  **your immediate family, or people you can confide in or talk to**  **about personal problems? (CF3)** | 9. Sind Sie der Meinung, dass es außerhalb Ihrer engsten Verwandtschaft niemanden gibt, dem Sie wirklich nahe stehen, oder dass es niemanden gibt, dem Sie vertrauen können oder mit dem Sie über persönliche Probleme reden können? | 0 | 1 | 2 | 3 | 4 |
| **I tend to keep my feelings to myself. (CA1)** | 10. Ich neige dazu, meine Gefühle für mich zu behalten. | 0 | 1 | 2 | 3 | 4 |
| **I rarely laugh and smile. (CA2)** | 11. Ich lache oder lächle selten. | 0 | 1 | 2 | 3 | 4 |
| **I am not good at expressing my true feelings by the way I talk**  **and look. (CA3)** | 12. Ich kann meine wahren Gefühle nicht gut durch meine Sprechweise und Mimik ausdrücken. | 0 | 1 | 2 | 3 | 4 |
| **Other people see me as slightly eccentric (odd). (EB1)** | 13. Andere Menschen halten mich für ein wenig seltsam. | 0 | 1 | 2 | 3 | 4 |
| **I am an odd, unusual person. (EB2)** | 14. Ich bin eine merkwürdige, ungewöhnliche Person. | 0 | 1 | 2 | 3 | 4 |
| **I have some eccentric (odd) habits. (EB3)** | 15. Ich habe ein paar exzentrische Gewohnheiten. | 0 | 1 | 2 | 3 | 4 |
| **People sometimes comment on my unusual mannerisms**  **and habits. (EB4)** | 16. Die Leute machen manchmal Bemerkungen über mein ungewöhnliches Gehabe und eigentümlichen Gewohnheiten. | 0 | 1 | 2 | 3 | 4 |
| **Do you often feel nervous when you are in a group of unfamiliar**  **people? (SA1)** | 17. Fühlen Sie sich oft angespannt, wenn Sie sich in einer Gruppe fremder Menschen befinden? | 0 | 1 | 2 | 3 | 4 |
| **I get anxious when meeting people for the first time. (SA2)** | 18. Wenn ich Menschen zum ersten Mal begegne, werde ich ängstlich. | 0 | 1 | 2 | 3 | 4 |
| **I feel very uncomfortable in social situations involving unfamiliar people. (SA3)** | 19. Unter Menschen, die ich nicht näher kenne, fühle ich mich sehr unwohl. | 0 | 1 | 2 | 3 | 4 |
| **I sometimes avoid going to places where there will be many people**  **because I will get anxious. (SA4)** | 20. Ich vermeide es manchmal, an Orte zu gehen, wo sich viele Menschen aufhalten, weil ich dort Angst bekomme. | 0 | 1 | 2 | 3 | 4 |
| **Do you believe in telepathy (mind-reading)? (MT1)** | 21. Glauben Sie an Gedankenübertragung? | 0 | 1 | 2 | 3 | 4 |
| **Do you believe in clairvoyance (psychic forces, fortune telling)?**  **(MT2)** | 22. Glauben Sie an das Hellsehen? | 0 | 1 | 2 | 3 | 4 |
| **Have you had experiences with astrology, seeing the future,**  **UFO’s, ESP, or a sixth sense? (MT3)** | 23. Hatten Sie bereits Erfahrungen mit Astrologie, Vorhersehen der Zukunft, UFOs, übersinnlicher Wahrnehmung oder dem sechsten Sinn? | 0 | 1 | 2 | 3 | 4 |
| **Have you ever felt that you are communicating with another person telepathically (by mind-reading)? (MT4)** | 24. Haben Sie jemals das Gefühl gehabt, mit einer anderen Person mittels Gedankenübertragung zu kommunizieren? | 0 | 1 | 2 | 3 | 4 |
| **I sometimes jump quickly from one topic to another when speaking.**  **(OS1)** | 25. Wenn ich spreche, springe ich manchmal schnell von einem Thema zum anderen. | 0 | 1 | 2 | 3 | 4 |
| **Do you tend to wander off the topic when having a conversation? (OS2)** | 26. Neigen Sie in einem Gespräch dazu, vom Thema abzukommen? | 0 | 1 | 2 | 3 | 4 |
| **I often ramble on too much when speaking. (OS3)** | 27. Beim Sprechen schweife ich oft zu sehr ab. | 0 | 1 | 2 | 3 | 4 |
| **I sometimes forget what I am trying to say. (OS4)** | 28. Ich vergesse manchmal, was ich gerade zu sagen versuche. | 0 | 1 | 2 | 3 | 4 |
| **I often hear a voice speaking my thoughts aloud. (UP1)** | 29. Ich höre oft eine Stimme meine Gedanken laut aussprechen. | 0 | 1 | 2 | 3 | 4 |
| **When you look at a person or yourself in a mirror, have you**  **ever seen the face change right before your eyes? (UP2)** | 30. Wenn Sie einen Menschen anschauen oder sich selbst im Spiegel betrachten, haben Sie jemals beobachtet, dass sich das Gesicht vor Ihren Augen verändert? | 0 | 1 | 2 | 3 | 4 |
| **Are your thoughts sometimes so strong that you can almost**  **hear them? (UP3)** | 31. Sind Ihre Gedanken manchmal so stark, dass Sie sie fast hören können? | 0 | 1 | 2 | 3 | 4 |
| **Do everyday things seem unusually large or small? (UP4)** | 32. Erscheinen alltägliche Gegenstände ungewöhnlich groß oder klein? | 0 | 1 | 2 | 3 | 4 |


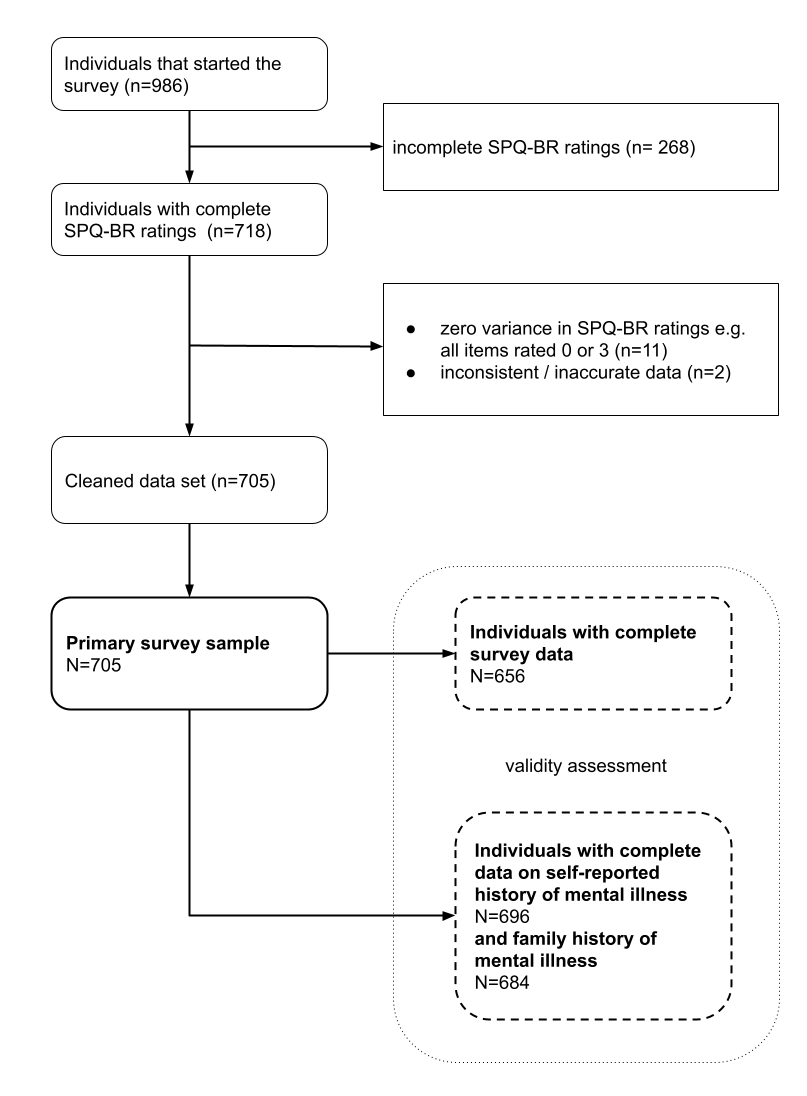


**Supplementary Figure 1**. Survey sample workflow and exclusion criteria


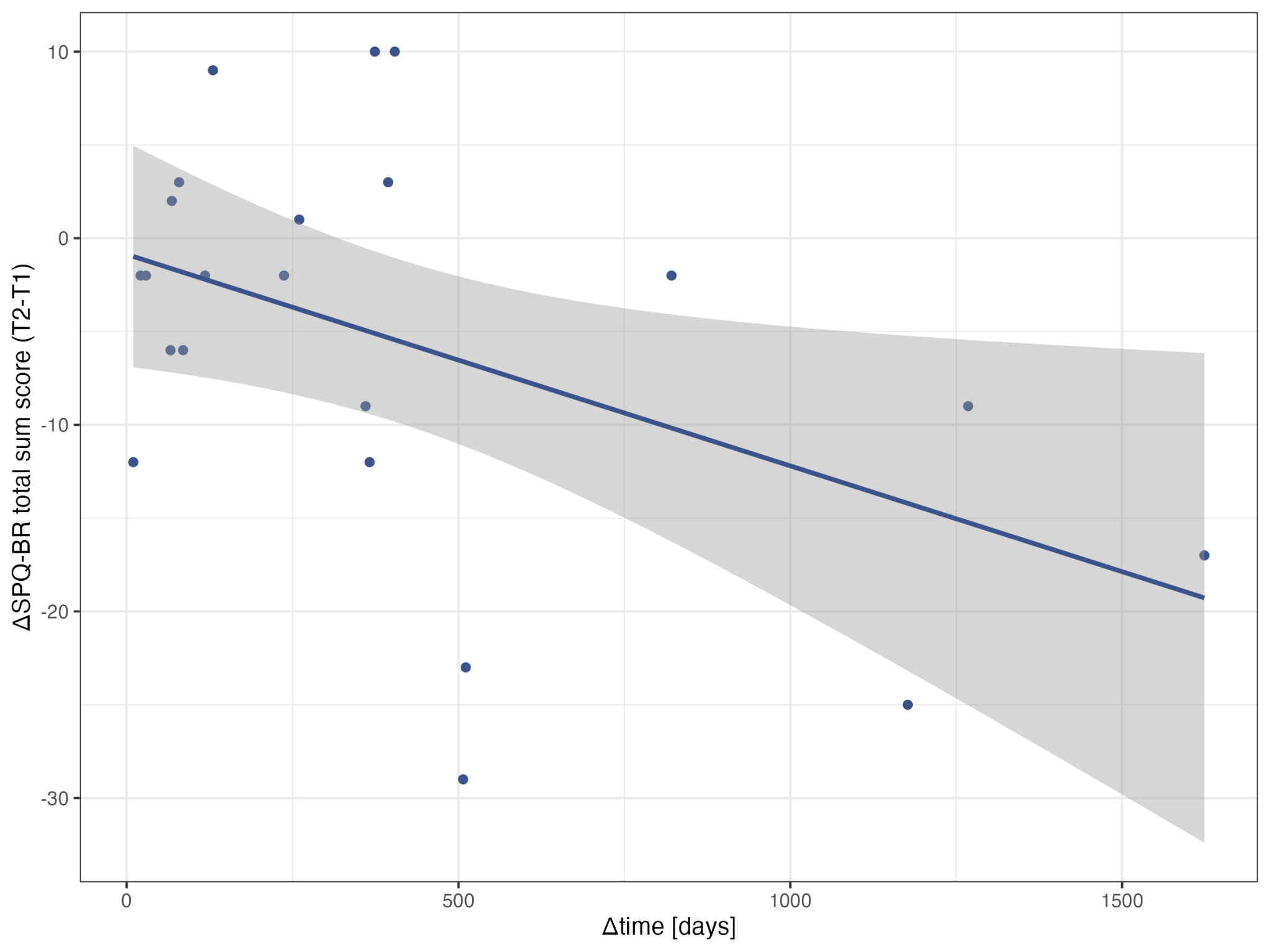
**Supplementary Figure 2.** Differences in SPQ-BR scores across time. Differences between the first and second SPQ-BR total sum scores (ΔSPQ-BR) are plotted as a function of the time interval between ratings (Δtime in days). Each dot represents one individual. The solid blue line depicts the fitted linear regression, and the grey shaded area indicates the 95% confidence interval.

**Supplementary Table 2**. Differences in SPQ-BR scores between male and female participants (N=703)

|  | **Male (N=164)^1^** | **Female (N=539)^1^** | **p-value*^2^*** |
| --- | --- | --- | --- |
| **SPQ-BR total** | 36.10 (18.30) | 35.94 (18.35) | 0.8 |
| **Superordinate factors** |  |  |  |
| Cognitive-perceptual deficits | 12.73 (8.73) | 13.44 (8.77) | 0.3 |
| Interpersonal deficits | 12.40 (7.35) | 12.20 (7.58) | 0.7 |
| Disorganisation | 10.97 (6.49) | 10.30 (6.28) | 0.2 |
| **Subordinate factors** |  |  |  |
| Ideas or reference and suspiciousness | 8.06 (4.88) | 8.18 (4.72) | 0.7 |
| Magical thinking | 2.50 (3.51) | 3.43 (3.82) | **<0.001** |
| Unusual perceptual experiences | 2.16 (3.01) | 1.82 (2.66) | 0.2 |
| Eccentric behavior | 4.69 (3.54) | 3.67 (3.53) | **<0.001** |
| Odd speech | 6.28 (4.14) | 6.63 (4.02) | 0.3 |
| Social anxiety | 4.96 (3.92) | 5.51 (4.08) | 0.12 |
| No close friends / constricted affect | 7.45 (4.68) | 6.70 (4.49) | 0.077 |

*^1^Mean (SD)*

*^2^Wilcoxon rank sum test*

**Supplementary Table 3**. Spearman correlations of SPQ-BR subordinate factors with the O-LIFE, SCL-90-R, NEO-FFI-30 & PHQ-4 scales (N=656)

| **Scale** | **IRS** | **MT** | **UP** | **CFCA** | **SA** | **OS** | **EB** |
| --- | --- | --- | --- | --- | --- | --- | --- |
| **O-LIFE** |  |  |  |  |  |  |  |
| Unusual Experience | 0.37^***^ | 0.53^***^ | 0.53^***^ | 0.17^***^ | 0.23^***^ | 0.36^***^ | 0.31^***^ |
| Introvertive Anhedonia | 0.20^***^ | 0.06 | 0.15^***^ | 0.49^***^ | 0.35^***^ | 0.19^***^ | 0.33^***^ |
| Cognitive Disorganization | 0.38^***^ | 0.21^***^ | 0.37^***^ | 0.37^***^ | 0.49^***^ | 0.47^***^ | 0.39^***^ |
| Impulsive Nonconformity | 0.22^***^ | 0.19^***^ | 0.27^***^ | 0.09^*^ | 0.18^***^ | 0.25^***^ | 0.28^***^ |
| **SCL-90-R** |  |  |  |  |  |  |  |
| Paranoid Ideation | 0.56^***^ | 0.29^***^ | 0.42^***^ | 0.37^***^ | 0.40^***^ | 0.35^***^ | 0.41^***^ |
| Psychoticism | 0.43^***^ | 0.24^***^ | 0.40^***^ | 0.45^***^ | 0.41^***^ | 0.34^***^ | 0.42^***^ |
| Schizophrenia Nuclear Symptoms | 0.25^***^ | 0.24^***^ | 0.38^***^ | 0.21^***^ | 0.20^***^ | 0.16^***^ | 0.25^***^ |
| Schizotypal Signs | 0.56^***^ | 0.28^***^ | 0.43^***^ | 0.45^***^ | 0.45^***^ | 0.38^***^ | 0.45^***^ |
| **NEO-FFI-30** |  |  |  |  |  |  |  |
| Neuroticism | 0.47^***^ | 0.19^***^ | 0.34^***^ | 0.42^***^ | 0.54^***^ | 0.39^***^ | 0.36^***^ |
| Extraversion | -0.10^*^ | 0.06 | -0.07 | -0.42^***^ | -0.41^***^ | 0.04 | -0.14^***^ |
| Openness to Experience | 0.01 | 0.07 | 0.11^**^ | -0.04 | 0.01 | 0.10^**^ | 0.16^***^ |
| Agreeableness | -0.30^***^ | -0.16^***^ | -0.30^***^ | -0.41^***^ | -0.27^***^ | -0.21^***^ | -0.47^***^ |
| Conscientiousness | -0.12^**^ | -0.06 | -0.19^***^ | -0.21^***^ | -0.14^***^ | -0.18^***^ | -0.31^***^ |
| **PHQ-4** |  |  |  |  |  |  |  |
| Anxiety | 0.33^***^ | 0.19^***^ | 0.30^***^ | 0.29^***^ | 0.42^***^ | 0.29^***^ | 0.29^***^ |
| Depression | 0.29^***^ | 0.15^***^ | 0.29^***^ | 0.36^***^ | 0.39^***^ | 0.27^***^ | 0.30^***^ |

*Note. *p < 0.05, **p < 0.01,***p < 0.001.*

*Abbreviations: O-LIFE = Oxford-Liverpool Inventory of Feelings and Experiences; SCL-90-R = Symptom Checklist 90 revised; NEO-FFI-30 = NEO Five-Factor Inventory; PHQ-4 = Patient Health Questionnaire 4*

*Abbreviations: IRS = Ideas of Reference / Suspiciousness; MT = Magical Thinking; UP = Unusual Perception; CF = No Close Friends; CA = constricted Affect; SA = Social Anxiety; EB = Eccentric Behaviour and OS = Odd Speech*

**Supplementary Table 4**. Association between SPQ-BR and personal wellbeing (PWI-A) (N=656)

|  | **SPQ-BR Total^1^** | **Cognitive-**  **Perceptual^1^** | **Interpersonal^1^** | **Disorganized^1^** | **IRS^1^** | **MT^1^** | **UP^1^** | **CFCA^1^** | **SA^1^** | **EB^1^** | **OS^1^** |
| --- | --- | --- | --- | --- | --- | --- | --- | --- | --- | --- | --- |
| **PWI-A mean score** | -0.43^***^  (-0.49,-0.36) | -0.61^***^  (-0.75,-0.47) | -1.07^***^  (-1.22,-0.92) | -0.91^***^  (-1.11,-0.72) | -1.10^***^  (-1.37,-0.84) | -0.77^***^  (-1.11,-0.42) | -1.50^***^  (-1.95,-1.04) | -1.65^***^  (-1.91,-1.40) | -1.61^***^  (-1.91,-1.32) | -1.72^***^  (-2.06,-1.38) | -0.89^***^  (-1.20,-0.57) |
| **living standard** | -0.33^***^  (-0.41,-0.25) | -0.54^***^  (-0.71,-0.38) | -0.73^***^  (-0.92,-0.54) | -0.70^***^  (-0.93,-0.47) | -0.91^***^  (-1.22,-0.60) | -0.75^***^  (-1.14,-0.36) | -1.46^***^  (-1.98,-0.93) | -1.19^***^  (-1.50,-0.87) | -1.03^***^  (-1.39,-0.68) | -1.41^***^  (-1.81,-1.01) | -0.61^***^  (-0.98,-0.25) |
| **health** | -0.37^***^  (-0.46,-0.28) | -0.54^***^  (-0.73,-0.34) | -0.84^***^  (-1.07,-0.62) | -0.88^***^  (-1.15,-0.61) | -1.00^***^  (-1.37,-0.64) | -0.58^**^  (-1.05,-0.11) | -1.43^***^  (-2.06,-0.81) | -1.16^***^  (-1.54,-0.79) | -1.45^***^  (-1.87,-1.03) | -1.38^***^  (-1.86,-0.90) | -1.07^***^  (-1.50,-0.64) |
| **life accomplishments** | -0.39^***^  (-0.47,-0.31) | -0.56^***^  (-0.73,-0.39) | -0.93^***^  (-1.12,-0.74) | -0.93^***^  (-1.16,-0.69) | -0.92^***^  (-1.24,-0.59) | -0.70^***^  (-1.12,-0.29) | -1.67^***^  (-2.22,-1.13) | -1.51^***^  (-1.84,-1.19) | -1.31^***^  (-1.68,-0.94) | -1.92^***^  (-2.33,-1.50) | -0.77^***^  (-1.16,-0.39) |
| **personal relationships** | -0.42^***^  (-0.51,-0.32) | -0.52^***^  (-0.72,-0.32) | -1.11^***^  (-1.33,-0.88) | -0.93^***^  (-1.21,-0.66) | -0.97^***^  (-1.34,-0.59) | -0.64^**^  (-1.11,-0.16) | -1.31^***^  (-1.94,-0.67) | -2.06^***^  (-2.42,-1.70) | -1.23^***^  (-1.66,-0.80) | -1.71^***^  (-2.19,-1.22) | -0.94^***^  (-1.38,-0.50) |
| **feeling secure** | -0.45^***^  (-0.53,-0.36) | -0.67^***^  (-0.86,-0.49) | -1.19^***^  (-1.40,-0.99) | -0.78^***^  (-1.04,-0.52) | -1.23^***^  (-1.57,-0.88) | -0.87^***^  (-1.31,-0.42) | -1.64^***^  (-2.24,-1.05) | -1.76^***^  (-2.11,-1.42) | -1.90^***^  (-2.29,-1.51) | -1.52^***^  (-1.98,-1.07) | -0.72^***^  (-1.14,-0.31) |
| **social participation** | -0.53^***^  (-0.61,-0.45) | -0.67^***^  (-0.86,-0.48) | -1.48^***^  (-1.68,-1.29) | -1.09^***^  (-1.35,-0.83) | -1.38^***^  (-1.73,-1.04) | -0.70^**^  (-1.15,-0.24) | -1.47^***^  (-2.07,-0.86) | -2.22^***^  (-2.55,-1.88) | -2.34^***^  (-2.72,-1.96) | -2.18^***^  (-2.63,-1.73) | -0.96^***^  (-1.38,-0.54) |
| **future security** | -0.50^***^  (-0.58,-0.41) | -0.73^***^  (-0.93,-0.54) | -1.20^***^  (-1.42,-0.98) | -1.07^***^  (-1.34,-0.80) | -1.32^***^  (-1.68,-0.95) | -1.13^***^  (-1.60,-0.67) | -1.50^***^  (-2.13,-0.87) | -1.67^***^  (-2.05,-1.30) | -2.04^***^  (-2.45,-1.63) | -1.92^***^  (-2.39,-1.44) | -1.13^***^  (-1.56,-0.69) |

*Note. Alle Werte sind signifikant **p < 0.01, ***p < 0.001.*

*Abbreviations: IRS = Ideas of Reference / Suspiciousness; MT = Magical Thinking; UP = Unusual Perception; CF = No Close Friends; CA = constricted Affect; SA = Social Anxiety; EB = Eccentric Behaviour and OS = Odd Speech*

*^1^beta (95% CI)*

**Supplementary Table 5**. Differences in SPQ-BR factor, subscale and item scores between individual with/without a family history of psychosis and mental disorders

|  | **Family history of psychosis** | | | **Family history of mental disorders** | | |
| --- | --- | --- | --- | --- | --- | --- |
|  | **Negative Family History, N=652^1^** | **Positive Family History, N=32^1^** | **p-value^2^** | **Negative Family History, N=483^1^** | **Positive Family History, N=201^1^** | **p-value^2^** |
| **SPQ-BR total** | 35.61 (18.04) | 35.94 (20.19) | >0.9 | 35.28 (17.58) | 36.43 (19.41) | 0.8 |
| **Superordinate factors** |  |  |  |  |  |  |
| Cognitive-perceptual deficits | 13.08 (8.56) | 13.91 (10.89) | >0.9 | 13.11 (8.46) | 13.12 (9.19) | 0.6 |
| Interpersonal deficits | 12.12 (7.51) | 11.78 (6.91) | >0.9 | 11.96 (7.36) | 12.45 (7.75) | 0.6 |
| Disorganisation | 10.41 (6.30) | 10.25 (7.00) | 0.8 | 10.21 (6.18) | 10.87 (6.67) | 0.3 |
| **Subordinate factors** |  |  |  |  |  |  |
| Ideas or reference and suspiciousness | 8.07 (4.69) | 8.06 (5.15) | 0.8 | 8.04 (4.64) | 8.15 (4.88) | >0.9 |
| Magical thinking | 3.15 (3.70) | 3.44 (4.96) | 0.6 | 3.24 (3.71) | 3.00 (3.91) | 0.2 |
| Unusual perceptual experiences | 1.85 (2.69) | 2.41 (3.43) | 0.7 | 1.84 (2.62) | 1.98 (2.98) | 0.6 |
| Eccentric behavior | 3.89 (3.55) | 3.62 (3.78) | 0.5 | 3.76 (3.44) | 4.14 (3.82) | 0.4 |
| Odd speech | 6.52 (4.03) | 6.62 (4.66) | >0.9 | 6.45 (4.03) | 6.72 (4.11) | 0.5 |
| Social anxiety | 5.29 (4.01) | 4.94 (4.16) | 0.6 | 5.23 (4.00) | 5.39 (4.06) | 0.6 |
| No close friends / constricted affect | 6.83 (4.56) | 6.84 (4.08) | 0.7 | 6.73 (4.43) | 7.05 (4.79) | 0.5 |

*^1^Mean (SD)*

*^2^Wilcoxon rank sum test*

**Supplementary table 6**. Differences in SPQ-BR factor, subscale and item scores between individual with/without schizophrenia or schizoaffective disorder and other self-reported mental disorders

|  | **Survey sample** | | | | **Survey vs. case sample** | | | |
| --- | --- | --- | --- | --- | --- | --- | --- | --- |
| **Characteristic** | **N** | **Negative self-reported history of mental illness, N=548^1^** | **Positive self-reported history of mental illness, N=148^1^** | **p-value^2^** | **N** | **sample 1 (survey),**  **N = 705^1^** | **sample 2 (cases),**  **N = 33^1^** | **p-value^2^** |
| Item 1 (IR4) | 696 | 1.65 (1.15) | 2.05 (1.28) | **<0.001** | 737 | 1.73 (1.19) | 2.25 (1.22) | **0.016** |
| Item 2 (IR5) | 696 | 1.36 (1.16) | 1.68 (1.26) | **0.005** | 738 | 1.43 (1.19) | 2.03 (1.36) | **0.010** |
| Item 3 (IR6) | 696 | 1.37 (1.10) | 1.57 (1.14) | 0.062 | 738 | 1.41 (1.11) | 2.00 (1.20) | **0.005** |
| Item 4 (S1) | 696 | 0.47 (0.81) | 0.80 (1.04) | **<0.001** | 738 | 0.54 (0.87) | 1.06 (1.12) | **0.001** |
| Item 5 (S2) | 696 | 1.44 (1.18) | 1.64 (1.22) | 0.070 | 738 | 1.48 (1.19) | 1.85 (1.28) | 0.10 |
| Item 6 (S3) | 696 | 1.51 (1.22) | 1.86 (1.30) | **0.004** | 738 | 1.59 (1.24) | 2.12 (1.43) | **0.030** |
| Item 7 (CF1) | 696 | 1.36 (1.08) | 1.54 (1.20) | 0.12 | 738 | 1.40 (1.11) | 2.03 (1.16) | **0.002** |
| Item 8 (CF2) | 696 | 1.09 (1.16) | 1.35 (1.28) | **0.034** | 738 | 1.15 (1.19) | 1.88 (1.39) | **0.002** |
| Item 9 (CF3) | 696 | 0.74 (1.08) | 0.85 (1.20) | 0.5 | 738 | 0.77 (1.11) | 1.52 (1.48) | **0.001** |
| Item 10 (CA1) | 696 | 1.91 (1.26) | 1.89 (1.29) | 0.9 | 738 | 1.91 (1.27) | 2.15 (1.28) | 0.3 |
| Item 11 (CA2) | 696 | 0.65 (0.89) | 0.77 (0.89) | **0.049** | 738 | 0.67 (0.89) | 1.42 (1.20) | **<0.001** |
| Item 12 (CA3) | 696 | 0.94 (1.06) | 1.11 (1.18) | 0.2 | 738 | 0.98 (1.08) | 1.55 (1.20) | **0.004** |
| Item 13 (EB1) | 696 | 1.05 (1.01) | 1.46 (1.23) | **<0.001** | 738 | 1.14 (1.08) | 2.45 (0.94) | **<0.001** |
| Item 14 (EB2) | 696 | 0.75 (0.92) | 1.23 (1.21) | **<0.001** | 738 | 0.85 (1.01) | 2.21 (1.08) | **<0.001** |
| Item 15 (EB3) | 696 | 0.99 (1.08) | 1.43 (1.29) | **<0.001** | 738 | 1.08 (1.14) | 1.85 (1.25) | **<0.001** |
| Item 16 (EB4) | 696 | 0.74 (0.94) | 1.14 (1.18) | **<0.001** | 738 | 0.83 (1.02) | 1.76 (1.20) | **<0.001** |
| Item 17 (SA1) | 696 | 1.64 (1.25) | 2.12 (1.28) | **<0.001** | 738 | 1.75 (1.27) | 2.73 (1.31) | **<0.001** |
| Item 18 (SA2) | 696 | 1.11 (1.10) | 1.56 (1.26) | **<0.001** | 738 | 1.22 (1.16) | 1.85 (1.28) | **0.005** |
| Item 19 (SA3) | 696 | 1.39 (1.15) | 1.72 (1.25) | **0.004** | 738 | 1.46 (1.18) | 2.21 (1.32) | **0.001** |
| Item 20 (SA4) | 696 | 0.82 (1.04) | 1.38 (1.31) | **<0.001** | 738 | 0.95 (1.13) | 1.67 (1.43) | **0.002** |
| Item 21 (MT1) | 696 | 1.11 (1.21) | 0.91 (1.16) | 0.064 | 738 | 1.06 (1.20) | 1.82 (1.47) | **0.002** |
| Item 22 (MT2) | 696 | 0.75 (1.04) | 0.86 (1.17) | 0.4 | 737 | 0.77 (1.06) | 1.19 (1.23) | **0.025** |
| Item 23 (MT3) | 696 | 0.63 (1.10) | 0.75 (1.26) | 0.5 | 738 | 0.66 (1.14) | 1.70 (1.59) | **<0.001** |
| Item 24 (MT4) | 696 | 0.73 (1.09) | 0.78 (1.19) | 0.9 | 738 | 0.74 (1.11) | 1.79 (1.62) | **<0.001** |
| Item 25 (OS1) | 696 | 1.79 (1.23) | 2.04 (1.26) | **0.033** | 738 | 1.85 (1.24) | 2.27 (1.35) | 0.056 |
| Item 26 (OS2) | 696 | 1.58 (1.15) | 1.90 (1.15) | **0.002** | 738 | 1.65 (1.15) | 2.18 (1.29) | **0.017** |
| Item 27 (OS3) | 696 | 1.34 (1.14) | 1.64 (1.17) | **0.004** | 738 | 1.39 (1.15) | 2.03 (1.33) | **0.005** |
| Item 28 (OS4) | 696 | 1.63 (1.18) | 1.72 (1.23) | 0.4 | 738 | 1.65 (1.19) | 2.30 (1.13) | **0.002** |
| Item 29 (UP1) | 696 | 0.47 (0.94) | 0.55 (1.04) | 0.5 | 738 | 0.49 (0.96) | 1.00 (1.37) | **0.032** |
| Item 30 (UP2) | 696 | 0.34 (0.80) | 0.46 (0.89) | **0.044** | 738 | 0.36 (0.82) | 0.85 (1.25) | **0.004** |
| Item 31 (UP3) | 696 | 0.71 (1.06) | 0.86 (1.22) | 0.3 | 738 | 0.74 (1.09) | 1.45 (1.30) | **<0.001** |
| Item 32 (UP4) | 696 | 0.30 (0.68) | 0.35 (0.76) | 0.7 | 738 | 0.31 (0.70) | 0.42 (0.71) | 0.2 |
| **SPQ-BR total** | 696 | 34 (17) | 42 (21) | **<0.001** | 736 | 36 (18) | 58 (20) | **<0.001** |
| **Superordinate factors** |  |  |  |  |  |  |  |  |
| Cognitive-perceptual | 696 | 13 (8) | 15 (10) | **0.022** | 736 | 13 (9) | 21 (9) | **<0.001** |
| Interpersonal | 696 | 12 (7) | 14 (8) | **<0.001** | 738 | 12 (8) | 19 (9) | **<0.001** |
| Disorganized | 696 | 10 (6) | 13 (7) | **<0.001** | 738 | 10 (6) | 17 (6) | **<0.001** |
| **Subordinate factors** |  |  |  |  |  |  |  |  |
| Ideas of Reference / Suspiciousness | 696 | 7.8 (4.6) | 9.6 (5.2) | **<0.001** | 737 | 8.2 (4.8) | 11.5 (5.8) | **<0.001** |
| Close Friends / Constricted Affect | 696 | 6.7 (4.4) | 7.5 (5.0) | 0.072 | 738 | 6.9 (4.5) | 10.5 (5.7) | **<0.001** |
| Eccentric Behaviour | 696 | 3.5 (3.2) | 5.3 (4.3) | **<0.001** | 738 | 3.9 (3.6) | 8.3 (3.3) | **<0.001** |
| Social Anxiety | 696 | 5.0 (3.9) | 6.8 (4.3) | **<0.001** | 738 | 5.4 (4.0) | 8.5 (4.6) | **<0.001** |
| Magical Thinking | 696 | 3.2 (3.7) | 3.3 (4.2) | 0.5 | 737 | 3.2 (3.8) | 6.4 (4.4) | **<0.001** |
| Odd Speech | 696 | 6.3 (4.0) | 7.3 (4.2) | **0.012** | 738 | 6.5 (4.1) | 8.8 (4.5) | **0.005** |
| Unusual Perceptions | 696 | 1.82 (2.61) | 2.23 (3.15) | 0.3 | 738 | 1.90 (2.74) | 3.73 (3.31) | **<0.001** |

*^1^Mean (SD); ^2^Wilcoxon rank sum test*

| **Supplementary Table 7**. SPQ-BR total sum score ranges per decile in the survey and clinical sample (n=736) | | |
| --- | --- | --- |
| **SPQ-BR decile** | **N** | **SPQ-BR sum score** |
| 1 (lowest) | 74 | ≤ 13 |
| 2 | 74 | 13 - 20 |
| 3 | 74 | 20 - 26 |
| 4 | 74 | 26 - 31 |
| 5 | 74 | 31 - 35 |
| 6 | 74 | 35 - 40 |
| 7 | 73 | 40 - 46 |
| 8 | 73 | 47 - 53 |
| 9 | 73 | 53 - 63 |
| 10 (highest) | 73 | ≥ 63 |

| **Supplementary Table 8.** Pairwise Spearman correlations between PANSS factor scores (Lim et al.) and SPQ-BR superordinate factors (N=33) | | | | | | | |
| --- | --- | --- | --- | --- | --- | --- | --- |
|  | SPQ-BR  (int) | SPQ-BR  (cog-per) | SPQ-BR  (dis) | PANSS  (pos) | PANSS  (neg) | PANSS  (cog-dis) | PANSS  (dep) |
| SPQ-BR (cog-per) | 0.47 |  |  |  |  |  |  |
| SPQ-BR (dis) | 0.69^**^ | 0.16 |  |  |  |  |  |
| PANSS (pos) | 0.49 | 0.53^*^ | 0.05 |  |  |  |  |
| PANSS (neg) | 0.86^***^ | 0.34 | 0.70^**^ | 0.44 |  |  |  |
| PANSS (cog-dis) | 0.09 | 0.17 | 0.09 | 0.44 | 0.15 |  |  |
| PANSS (dep) | 0.44 | 0.53^*^ | 0.49 | 0.34 | 0.24 | -0.05 |  |
| PANSS (hos) | 0.26 | -0.20 | 0.54^*^ | -0.09 | 0.20 | -0.05 | 0.20 |
| *Note. *p < 0.05, **p < 0.01,***p < 0.001.*  *Abbreviations: PANSS = Positive and Negative Syndrome Scale; int = Interpersonal; dis - Disorganized; cog-per = Cognitive-Perceptual; pos = positive; neg = negative; cog-dis = cognitive / disorganized; dep = depressive; hos = hostility;* | | | | | | | |

| **Supplementary Table 9.** Sociodemographic characteristics of the independent, longitudinal sample (n=22) | | |
| --- | --- | --- |
|  | **Timepoint** | |
| **Characteristic** | **T1**, N = 22^1^ | **T2**, N = 22^1^ |
| **Gender (female)** | 21 (95%) | - |
| **Age** | 23.0 (20.3, 30.8) | 25.0 (22.0, 31.0) |
| **Native Language (German)** | 22 (100%) | - |
| **Education (ISCED-97)** |  |  |
| upper secondary | 11 (50%) | 10 (45%) |
| first stage tertiary | 11 (50%) | 12 (55%) |
| **Employment** |  |  |
| In training / Studying | 16 (73%) | 15 (71%) |
| Employed, full-time or part-time | 7 (32%) | 9 (43%) |
| **Negative history of mental illness** | 21 (95%) | 20 (91%) |
| **Relationship Status** |  |  |
| In a relationship/married | 15 (68%) | 16 (73%) |
| Single/divorced/widowed | 7 (32%) | 6 (27%) |
| **Housing** |  |  |
| Flatshare | 8 (36%) | 10 (45%) |
| Living alone | 3 (14%) | 3 (14%) |
| Living with family members | 11 (50%) | 9 (41%) |
| ^1^n (%); Median (IQR) | | |

| **Supplementary Table 10.** Differences in SPQ-BR scores across time | | | | | | |
| --- | --- | --- | --- | --- | --- | --- |
|  |  | **T1 (first rating)** | | **T2 (second rating)** | | **T2-T1** |
| **Group^1^** | **N** | **Mean (SD)** | **Range** | **Mean (SD)** | **Range** | **Mean (SD)** |
| all | 22 | 26.27 (14.26) | 2-59 | 20.82 (14.41) | 0-53 | -5.46 (10.86) |
| ≤ 1 year | 12 | 22.75 (17.45) | 2-59 | 20.58 (15.49) | 0-53 | -2.17 (5.65) |
| > 1 year | 10 | 30.50 (8.13) | 12-38 | 21.10 (13.81) | 3-41 | -9.40 (14.29) |
| *Note.* **^1^***Group: all participants, participants who rated the SPQ-BR within one year, and participants where SPQ-BR ratings occurred more than one year apart.* | | | | | | |

**Supplementary References**

1. [Braun, A., Kraft, J. & Ripke, S. Study protocol of the Berlin Research Initiative for Diagnostics, genetics and Environmental Factors in schizophrenia (BRIDGE-S). *BMC Psychiatry* **23**, 31 (2023).](http://paperpile.com/b/2iIoZC/iB9s)

2. [Koo, T. K. & Li, M. Y. A guideline of selecting and reporting intraclass correlation coefficients for reliability research. *J. Chiropr. Med.* **15**, 155–163 (2016).](http://paperpile.com/b/2iIoZC/gFog)

3. [Geng, F.-L. *et al.* Developmental trajectories of schizotypal personality disorder-like behavioural manifestations: a two-year longitudinal prospective study of college students. *BMC Psychiatry* **13**, 323 (2013).](http://paperpile.com/b/2iIoZC/zDe9)

4. [Karamaouna, P., Zouraraki, C. & Giakoumaki, S. G. Cognitive functioning and schizotypy: A four-years study. *Front. Psychiatry* **11**, 613015 (2020).](http://paperpile.com/b/2iIoZC/vZ1i)

5. Klein, C., Andresen, B. & Jahn, T. Erfassung der schizotypen Persönlichkeit nach DSM-III-R: Psychometrische Eigenschaften einer autorisierten deutschsprachigen Übersetzung des‘ Schizotypal Personality Questionnaire’(SPQ) von Raine. Diagnostica (1997).
